# In vivo evaluation of GG2–GG1/A2 element activity in the insulin promoter region using the CRISPR–Cas9 system

**DOI:** 10.1038/s41598-021-99808-6

**Published:** 2021-10-13

**Authors:** Hirofumi Noguchi, Chika Miyagi-Shiohira, Takao Kinjo, Issei Saitoh, Masami Watanabe

**Affiliations:** 1 Department of Regenerative Medicine, Graduate School of Medicine, University of the Ryukyus, Nishihara, Okinawa 903-0215 Japan; 2 Department of Basic Laboratory Sciences, School of Health Sciences in Faculty of Medicine, University of the Ryukyus, Nishihara, Okinawa 903-0215 Japan; 3 Department of Pediatric Dentistry, Asahi University School of Dentistry, Hozumi, Mizuho, 501-0296 Japan; 4 Department of Urology, Okayama University Graduate School of Medicine, Dentistry and Pharmaceutical Sciences, Okayama, Okayama 700-8558 Japan

**Keywords:** Biological techniques, Cell biology

## Abstract

The insulin promoter is regulated by ubiquitous as well as pancreatic β-cell-specific transcription factors. In the insulin promoter, GG2–GG1/A2–C1 (bases − 149 to − 116 in the human insulin promoter) play important roles in regulating β-cell-specific expression of the insulin gene. However, these events were identified through in vitro studies, and we are unaware of comparable in vivo studies. In this study, we evaluated the activity of GG2–GG1/A2 elements in the insulin promoter region in vivo. We generated homozygous mice with mutations in the GG2–GG1/A2 elements in each of the *Ins1* and *Ins2* promoters by CRISPR–Cas9 technology. The mice with homozygous mutations in the GG2–GG1/A2 elements in both *Ins1* and *Ins2* were diabetic. These data suggest that the GG2–GG1/A2 element in mice is important for *Ins* transcription in vivo.

## Introduction

The promoter of the insulin gene (*Ins*) consists of DNA sequences immediately upstream of the site of transcription initiation^1–5^. In the insulin promoter, three highly conserved enhancer elements, A3 (bases − 225 to − 220 in the human insulin promoter)^6–9^, GG2–GG1/A2–C1 (bases − 149 to − 116)^10^ and E1 (bases − 111 to − 102)^11,12^, play important roles in regulating β-cell-specific expression of the insulin gene. In the human insulin gene, GG elements with the consensus core sequence of GGAAAT, such as GG2 at bases − 145 to − 140 and GG1 at bases − 134 to − 129, are located immediately upstream of C1^13^. GG1 is also identified as A2 according to simplified nomenclature^14^. The GG1/A2 element overlaps with the C1 element^14^, and this region in the rat *insulin II* gene (*Ins2*) has been studied in detail^10,12,15^. The GG1/A2–C1 region, which is also called rat insulin promoter element 3b (RIPE3b), works together with the nearby E1 element, and this synergy is dependent on the C1 element^15^. The V-maf musculoaponeurotic fibrosarcoma oncogene family protein A (MafA) binds to the C1 element^16^. The A2.2 factor, which is a β-cell-specific activator that has not yet been identified, binds to the A2 element^17^. The MafA and A2.2 factors cooperatively activate insulin gene expression^18^.

The GG2 element contributes to the β-cell-specific transcription of the human insulin gene^13,19^, and it is a site for pancreatic and duodenal homeobox1 (PDX1) activation in the human insulin gene^20^. In contrast, the rodent GG2 element is negatively regulated by the Nkx2.2 transcription factor, although the sequences surrounding human GG2 (GGAAAT) and rat GG2 are only different at nucleotides − 144 and − 141 (*Insulin I* (*Ins1*); GCAAGT) or nucleotide − 141 (*Ins2*; GGAAGT) in humans denoted^20,21^. In the human GG element, the nucleotide − 141 mutant (GGAAAT → GGAAGT) exhibited decreased activity from the insulin promoter, while the nucleotide − 144 mutant (GGAAAT → GCAAAT) resulted in 3–4-fold activation over the wild type^20,21^. However, these events were identified through in vitro studies, and we are unaware of comparable in vivo studies.

MafA-deficient mice display intolerance to glucose and develop diabetes mellitus. *Ins1* and *Ins2* transcripts are diminished in MafA-deficient mice^22^. Mice lacking Pdx1 fail to form a pancreas^23^, and β-cell-specific inactivation of the mouse Pdx1 gene results in loss of the β-cell phenotype and maturity-onset diabetes^24^. Nkx2.2-deficient mice develop severe hyperglycemia and die shortly after birth^25^. These data show that MafA, Pdx1, and Nkx2.2 are important for pancreas development and that MafA affects the *Ins1* and *Ins2* transcripts in vivo. However, it is unknown whether the GG2–GG1/A2 sequence in insulin promoters is important for *Ins1* and *Ins2* transcripts in vivo because there are several binding sites of MafA and Pdx1 in insulin promoters and the three transcription factors affect pancreas development.

In this study, we evaluated the activity of GG2–GG1/A2 elements of the insulin promoter region in vivo. Clustered regularly interspaced short palindromic repeat (CRISPR)-Cas9 technology was used to generate mice with partial deletions of the *Ins1* and *Ins2* promoters, and the results showed that only homozygous mice with mutations in the highly conserved GG2–GG1/A2 elements of the *Ins1* and *Ins2* promoters developed diabetes*.*

## Results

### Generation of mice with insulin promoter mutations

**Figure 1:**
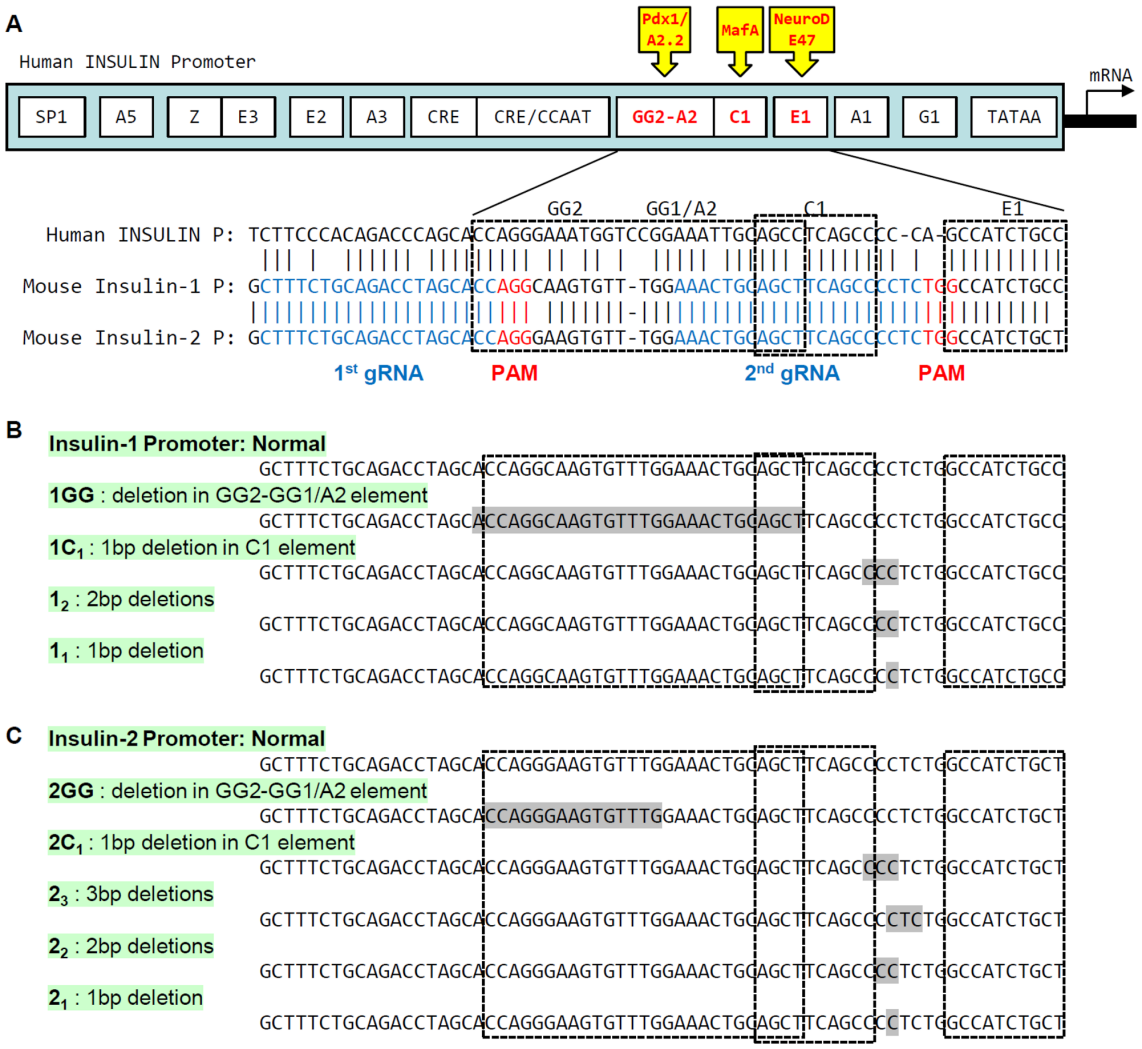
Generation of mice with mutations of the insulin promoter. (**A**) Structures and sequences of the human and mouse insulin promoters. Bases − 170 to − 147 and − 145 to − 104 of the mouse *Ins1* and *Ins2* promoters are identical, and bases − 149 to − 147 (AGG) and − 114 to − 112 (TGG) were used as the PAM sequences of the guide RNAs (gRNAs) for the CRISPR–Cas9 system. The 20-bp double-stranded DNAs (dsDNAs) derived from positions − 169 to − 150 and − 134 to − 115 of the *Ins1/2* promoters were inserted into pX330. (**B**, **C**) Generation of mice with mutated insulin promoters. The mice with 4 types of deletions in the *Ins1* promoter (**B**) and 5 types of deletions in the *Ins2* promoter (**C**) were generated. Sequences shaded in gray were deleted.

**Figure 2:**
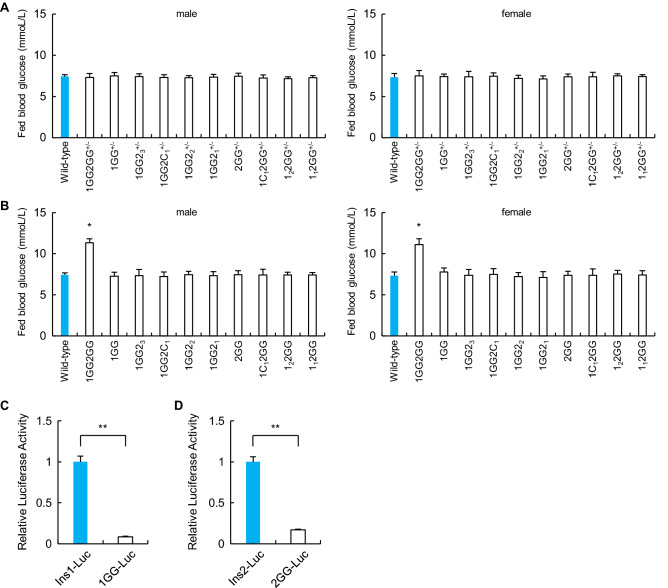
Blood glucose levels of mice with mutations of the insulin promoter and luciferase assay results. (**A**) Blood glucose levels of fed wild-type (n = 6) and heterozygous mice that had deletions in the *Ins1/2* promoters (n = 6 each) at 12 weeks of age. (**B**) Blood glucose levels of fed wild-type (n = 6) and homozygous mice that had deletions in the *Ins1/2* promoters (n = 6 each) at 12 weeks of age. (**C**) Mouse *Ins1* promoter-luciferase plasmid containing approximately 1000 bp of the 5′-flanking sequences of the wild-type or mutated *Ins1* promoter region was transfected into βTC6 cells (β-cell line). Forty-eight hours after transfection, cells were harvested and assayed (n = 3). **p < 0.01. (**D**) Mouse *Ins2* promoter-containing luciferase plasmid containing approximately 1000 bp of the 5′-flanking sequences of the wild-type or *Ins2* deletion promoter region was transfected into βTC6 cells (β-cell line). Forty-eight hours after transfection, cells were harvested and assayed (n = 3). **p < 0.01. The error bars represent the standard error.

We previously reported the generation of mice with mutations in C1 elements of the *Ins1* and *Ins2* genes using CRISPR–Cas9 technology^26^. We comicroinjected the same pX330 vectors to express two guide RNAs (gRNAs) (Fig. 1A) and Cas9 (pX330-1st/2nd gRNA vectors) in mouse zygotes^27–30^. Among the neonates, we detected mutations in the GG2–GG1/A2 elements of the *Ins1* and *Ins2* promoters (Fig. 1B,C). We also analyzed mutations in 3 and 4 other sequences bordering the C1 elements of the *Ins1* and *Ins2* promoters, respectively (Fig. 1B,C). To evaluate the importance of the GG2–GG1/A2 elements in vivo, mice with 4 and 5 of these mutations in the *Ins1* and *Ins2* promoters, respectively, were crossed with wild-type C57BL/6J mice. Nine heterozygous mice that had deletions in the *Ins1* or *Ins2* promoter were generated, and they were not diabetic (Fig. 2A, Supplementary Table 1). The F1 mice were intercrossed to obtain homozygous F2 mice that had deletions in only the *Ins1* promoter or the *Ins2* promoter. These mice were also not diabetic (Fig. 2B, Supplementary Table 2).

The homozygous F2 mice that had deletions in only the *Ins1* promoter or the *Ins2* promoter were intercrossed to obtain heterozygous F3 mice that had deletions in both the *Ins1* promoter and the *Ins2* promoter. These mice were not diabetic (Fig. 2A, Supplementary Table 3). Twenty homozygous mice that had deletions in both the *Ins1* and *Ins2* promoters were obtained by crossing the F3 and F4 mice (Supplementary Table 4). Only homozygous mice with mutations in the GG2–GG1/A2 elements of both the *Ins1* and *Ins2* promoters (1GG2GG mice) were diabetic (Fig. 2B). Insulin promoter activity was significantly decreased for the 2 types of mutations in the *Ins1* and *Ins2* promoters (Fig. 2C,D).

### Immunohistochemistry of the pancreatic tissue of 1GG2GG mice

**Figure 3:**
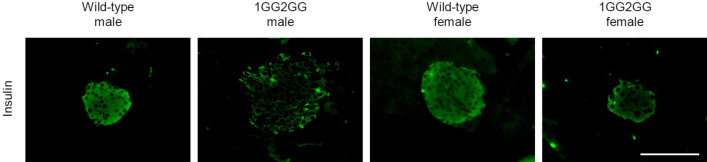
Immunohistochemistry. Immunohistochemical analysis of pancreatic islets (insulin) in wild-type and 1GG2GG mice. Scale bars = 100 µm.

Immunohistochemical analysis of the pancreatic tissue of both 1GG2GG male and female mice showed reduced insulin expression in islets compared with what was observed in the islets of wild-type C57BL/6 mice (Fig. 3).

### Blood glucose levels of 1GG2GG mice

**Figure 4:**
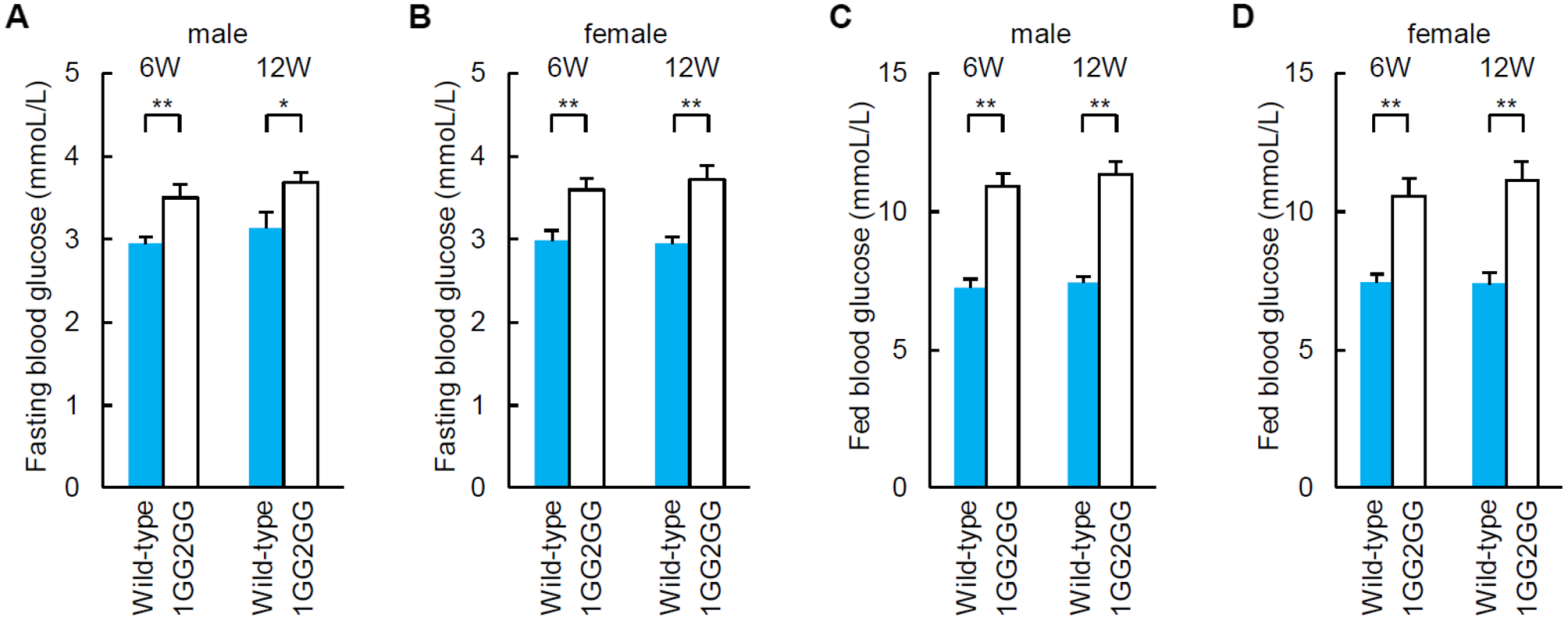
Blood glucose levels of 1GG2GG mice. (**A**, **B**) Fasting blood glucose concentrations of wild-type (n = 6) and 1GG2GG mice (n = 6) at 6 and 12 weeks of age [(**A**): male, (**B**): female]. (**C**, **D**) Blood glucose levels of fed wild-type (n = 6) and 1GG2GG mice (n = 6) at 6 and 12 weeks of age [(**C**): male, (**D**): female]. *p < 0.05. **p < 0.01.

Fasted blood glucose levels of both male and female 1GG2GG mice at 6 weeks of age were elevated compared with those of wild-type C57BL/6 mice (Fig. 4A, B). Fed blood glucose levels of both male and female 1GG2GG mice at 6 weeks of age were also elevated compared with those of wild-type C57BL/6 mice (Fig. 4C, D). Blood glucose levels of fasted and fed 1GG2GG mice at 12 weeks of age were elevated compared with those of wild-type C57BL/6 mice (Fig. 4A–D).

### mRNA levels in pancreatic islets of 1GG2GG mice

**Figure 5:**
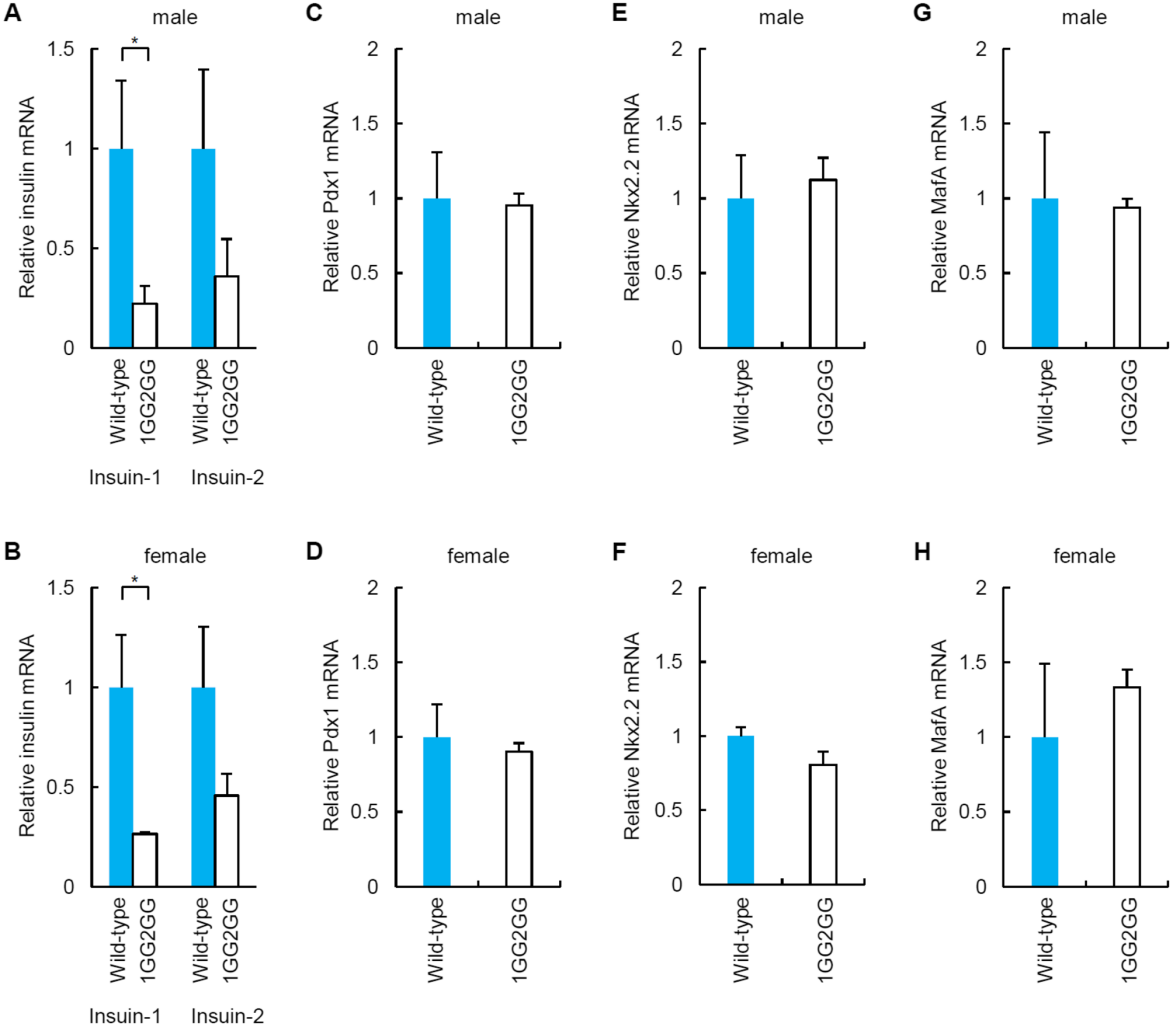
*Ins1/2, Pdx1*, *Nkx2.2,* and *MafA* mRNA levels in pancreatic islets from 1GG2GG mice. (**A**, **B**) qRT-PCR analysis of *Ins1* and *Ins2* in pancreatic islets of 1GG2GG mice [(**A**): male, (**B**): female]. (**C**, **D**) qRT-PCR analysis of *Pdx1* expression in pancreatic islets of 1GG2GG mice [(**C**): male, (**D**): female]. (**E**, **F**) qRT-PCR analysis of *Nkx2.2* expression in pancreatic islets of 1GG2GG mice [(**E**): male, (**F**): female]. (**G**, **H**) qRT-PCR analysis of *MafA* expression in pancreatic islets of 1GG2GG mice [(**G**): male, (**H**): female]. Pancreatic islets (purity > 95%) of wild-type mice served as a control. The data are expressed as the gene-to-*Gapdh* ratio; that of the control cells was arbitrarily defined as 1 (n = 8). The error bars represent the standard error. *p < 0.05.

Quantitative RT-PCR analysis of mRNA extracted from the pancreatic islets of male and female 1GG2GG mice (fasted) with diabetes showed that *Ins1* and *Ins2* mRNA levels were decreased compared with those of wild-type C57BL/6 mice (fasted) (Fig. 5A,B). There were no significant differences in the levels of the following mRNAs: *Pdx1*, *Nkx2.2*, and *MafA* (Fig. 5C–H). We also evaluated *Ins1* and *Ins2* mRNA levels in the embryonic pancreas of 1GG2GG mice. There were no significant differences between *Ins1* and *Ins2* mRNA levels in the embryonic pancreas of 1GG2GG mice and that of C57BL/6 mice (Supplementary Fig. 1).

### Glucose tolerance tests and body weight of 1GG2GG mice

**Figure 6:**
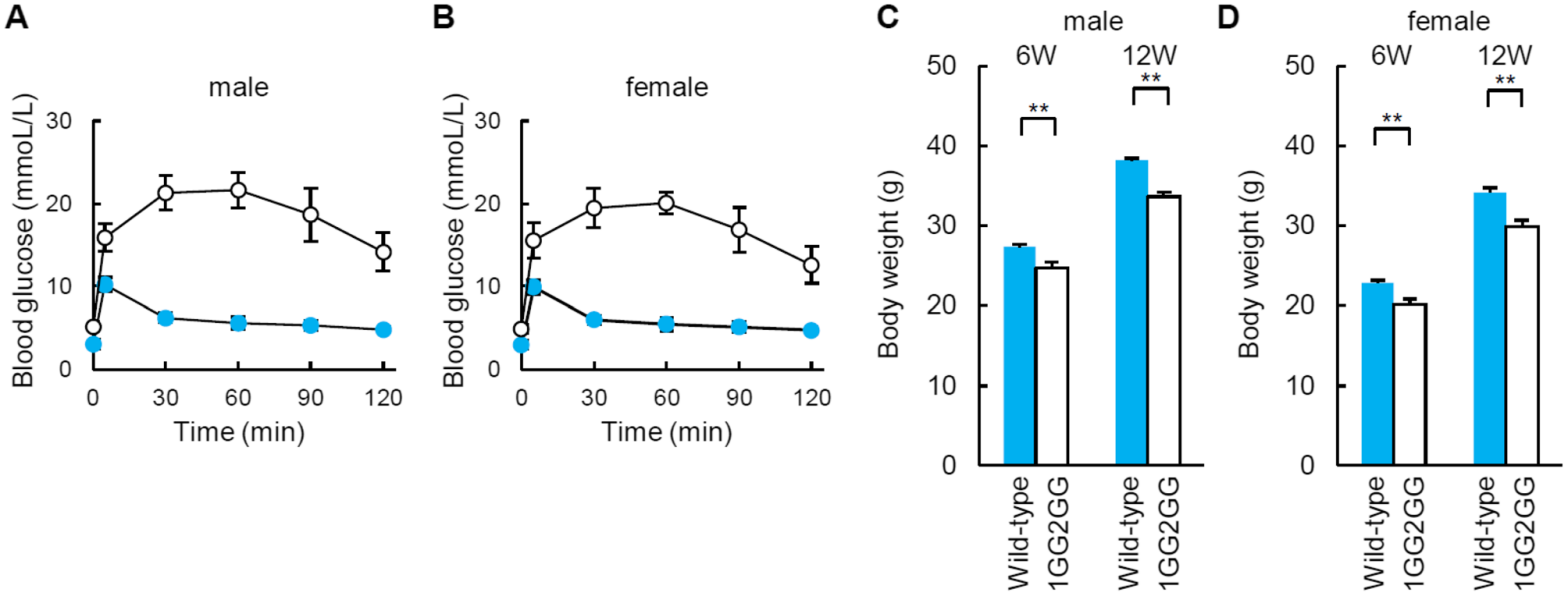
Glucose tolerance test and body weights of 1GG2GG mice. (**A**, **B**) Intraperitoneal glucose tolerance testing of wild-type (blue: n = 6) and 1GG2GG mice (white: n = 6) at 12 weeks of age [(**A**): male, (**B**): female]. (**C**, **D**) Body weights of wild-type (n = 6) and 1GG2GG mice (n = 6) at 6 and 12 weeks of age [(**C**): male, (**D**): female]. The error bars represent the standard error. **p < 0.01.

Glucose tolerance tests showed significantly elevated glucose levels in male and female 1GG2GG mice (Fig. 6A,B). Both male and female 1GG2GG mice exhibited decreased body weights at 6 and 12 weeks (Fig. 6C,D).

1GG2GG mice with mutations in the highly conserved GG2–GG1/A2 elements of the *Ins1* and *Ins2* promoters developed diabetes, and the body weight of 1GG2GG mice was lower than that of wild type controls. However, 20–30% of insulin transcription in 1GG2GG mice remained, and they were fertile.

## Discussion

Here, we used the CRISPR–Cas9 system to evaluate the functions of the promoter regions of *Ins1* and *Ins2* in vivo. The use of CRISPR–Cas9 systems to manipulate mammalian genomes presents enormous opportunities for curing human diseases^28–32^. The nonhomologous end joining (NHEJ) mechanism induces site-specific repair at the DNA break site, which causes different and unpredictable insertions or deletions of various sizes. Although the NHEJ features of the CRISPR–Cas9 system are disadvantageous for the generation of knockout mice via the deletion of a single protein-encoding gene, the system is advantageous for evaluating the functions of promoter regions in vivo because multiple mice with different alterations of promoter sequences can be generated concurrently.

We generated diabetic mice with deletions in the GG2–GG1/A2 elements of both the *Ins1* and *Ins2* promoters. Insulin promoter activity was significantly decreased for the 2 types of *Ins1* and *Ins2* promoters (Fig. 2C,D). Although the deletions in the GG2–GG1/A2 elements of the *Ins1* promoters were complete, the deletions in the *Ins2* promoter mainly involved the GG2 elements. In the human insulin gene, the GG2 element is a positive regulator of β-cell-specific transcription^13,19^. In contrast, it has been reported that the rodent GG2 element is negatively regulated by the Nkx2.2 transcription factor^20,21^. The sequence surrounding human GG2 is GGAAAT, and in mice, the GG2 is GCAAGT in *Ins1* and GGAAGT in *Ins2*. In our study, the sequence of the deletions in the *Ins2* promoter was CCAGGGAAGTGTTTG, and the deletions resulted in a decrease in insulin promoter activity. The sequences bordering the GG2 element (CCAG or GTTTG) may be important for insulin promoter activity.

Homozygous mice with deletions in only the *Ins1* promoter or the *Ins2* promoter were not diabetic (Fig. 2B). In the single mutant models, compensation of *Ins1*/*Ins2* was demonstrated (Supplementary Fig. 2). Our previous study showed that compensatory transcription of a functional insulin gene in homozygous mice with deletions of the C1 element in either the *Ins1* or *Ins2* promoter did not lead to diabetes^26^. Moreover, it has been reported that mice with single homozygous null mutations in *Ins1* or *Ins2* were not diabetic because of compensatory responses, and there was a dramatic increase in *Ins1* transcripts in the *Ins2*^−/−^ mice^33^. Compensatory transcription results from nondiabetic mice with homozygous deletions in only the *Ins1* promoter or the *Ins2* promoter.

Although the overlapping sequence of the C1 element (AGCT) is deleted in 1GG of the insulin-1 promoter, the overlapping sequence of the C1 element is not deleted in 2GG of the insulin-2 promoter. 1GG2GG mice become diabetic, while 1GG mice do not become diabetic, suggesting that the deletion of the GG2–GG1/A2 element excluding the overlapping sequence of the C1 element in the insulin-2 promoter (CCAGGGAAGTGTTTG) is important for the positive regulation of the insulin-2 gene. Our data are the first to report that the CCAGGGAAGTGTTTG sequence in the insulin-2 promoter is important in vivo. On the other hand, 1GG2GG mice develop diabetes, while 2GG mice do not develop diabetes, suggesting that the deletion of the GG2–GG1/A2 element, including the overlapping sequence of the C1 element in the insulin-1 promoter (ACCAGGCAAGTGTTTGGAAACTGCAGCT), is important for the positive regulation of the insulin-1 gene in vivo. We cannot decide whether the CCAGGCAAGTGTTTG sequence or the overlapping AGCT sequence or both is important for the insulin-1 promoter.

The effect on fed and fasting glucose levels in 1GG2GG mice was very mild (Fig. 2B) compared to the inactivity of insulin-driven mutant luciferase activity (Fig. 2C,D), reduced insulin transcription (Fig. 5A,B), and the degree of glycemic excursion by glucose tolerance test (Fig. 6). This may be due to the remaining capacity of the pancreas. Living donor segmental pancreas transplants have been performed clinically, and most of the donors did not develop diabetes, at least not immediately after donation^34^. Half of the pancreas was donated at that time, but it did not induce diabetes, suggesting that pancreata have remaining capacity. Although insulin transcription is severely reduced in 1GG2GG mice, the degree of diabetes is mild, probably due to the remaining capacity of the pancreas.

In conclusion, the sequences of the GG2–GG1/A2 elements in the *Ins1* and *Ins2* promoters are required for *Ins* expression in vivo. To the best of our knowledge, our findings show for the first time that the GG2–GG1/A2 elements of the insulin promoter in mice are important for its activity in vivo. The CRISPR–Cas9 technique will provide a tool to generate knockout mice that can be used to evaluate promoter regions.

## Methods

### Generation of mice with insulin promoter mutations

We previously constructed two Cas9-single-guide RNA (sgRNA) expression vectors^26^. The 1st gRNA and 2nd gRNA were inserted into pX330 vectors^35^ (Addgene, Watertown, MA). Female C57BL/6J mice were injected with pregnant mare serum gonadotropin and human CG (hCG) at 48-h intervals and then were mated with male C57BL/6J mice. Fertilized one-cell embryos were collected from the oviducts. Subsequently, 5 ng/μL pX330-1st/2nd gRNA vectors were injected into the pronuclei of these one-cell embryos, which were transferred into pseudopregnant mice from the Institute of Cancer Research (ICR). F0 mice were genotyped to detect the presence of mutations in the *Ins1/2* promoters. F0 mice were checked for the Cas9 transgene and for off-target effects^36^. F0 mice were mated with C57BL/6J mice to obtain F1 offspring.

The mutant mice and littermate wild-type mice were maintained on a C57BL/6 background. Mice were housed under a 12:12-h light/dark cycle in a room with controlled temperature and humidity. Food and water were provided without restrictions^26^. The Institutional Animal Care and Use Committee of the University of the Ryukyus and the University of Tsukuba approved the animal studies.

#### Construction of insulin promoter-luciferase plasmids

Wild-type or mutated *Ins1/2* promoter cDNAs containing approximately 1000 bp of the 5′-flanking sequences of the *Ins1/2* promoter regions were amplified using PCR with appropriate linker-containing primers, which were then used to replace the promoter region of the Rat *Ins2* promoter–reporter (luciferase) plasmid^37^ using a ligation kit (TaKaRa, Tokyo, Japan) (Supplementary Fig. 3).

#### Reporter assay

βTC6 cells (β-cell line) (ATCC, Manassas, VA) were transfected using Lipofectamine (Thermo Fisher Scientific, Tokyo, Japan) according to the conditions recommended by the manufacturer. The cells were transfected with 1.0 μg of wild-type or mutated reporter (luciferase) plasmids containing the *Ins1* or *Ins 2* promoter in 35-mm culture dishes. Forty-eight hours after transfection, the cells were harvested and assayed (Promega, Madison, WI).

#### Immunofluorescence staining

A guinea pig anti-insulin antibody (Abcam, Tokyo, Japan) and a FITC-conjugated anti-guinea pig IgG (Abcam) were used for immunofluorescence staining of mouse pancreatic islets. Pancreatic tissues were fixed with 4% paraformaldehyde in phosphate-buffered saline (PBS). Paraffin sections were mounted on slides. After blocking with 20% AquaBlock (EastCoast Bio, North Berwick, ME, USA) for 30 min at room temperature, the sections were incubated overnight at 4 °C with a guinea pig anti-insulin antibody (1:100); then, they were incubated for 1 h at room temperature with a FITC-conjugated anti-guinea pig IgG (1:250).

#### Islet isolation from mouse pancreas

Islets were isolated from the pancreata of normal and genome-edited mice^38,39^. For islet isolation, the common bile duct was cannulated and injected with cold Hank’s balanced salt solution (HBSS, Thermo Fisher Scientific) containing 1.5 mg/mL collagenase (Roche Boehringer Mannheim, Indianapolis, IN). The pancreas was digested at 37 °C. The islets were separated with a Histopaque 1077 (Merck) density gradient; then, they were hand-picked using a dissecting microscope to ensure pure islet preparation and were used immediately.

#### Quantitative RT-PCR analysis of isolated islets

Total RNA was extracted from isolated islets using an RNeasy Mini Kit (Qiagen, Tokyo, Japan). After quantifying the RNA using spectrophotometry, 2.5 µg of RNA was heated at 85 °C for 3 min and then reverse-transcribed in a 25-µL reaction mixture containing 200 units of Superscript II RNase H-RT (Thermo Fisher Scientific), 50 ng random hexamers (Thermo Fisher Scientific), 160 µmol/l dNTP and 10 nmol/l dithiothreitol. The reaction mixture was incubated for 10 min at 25 °C, 60 min at 42 °C and 10 min at 95 °C^26^.

Quantitative PCR amplification of cDNA from mouse islets was performed using a TaqMan universal PCR master mix core reagent kit according to the manufacturer’s instructions (Applied Biosystems, Foster City, CA, USA). PCR was performed for 40 cycles, and the reactions were incubated for 2 min at 50 °C and 10 min at 95 °C for the initial steps. In each cycle, denaturation was performed for 15 s at 95 °C, and annealing/extension was performed for 1 min at 60 °C. PCR was performed in 20 µL of solution using cDNAs synthesized from 1.11 ng of total RNA. The amount of mRNA was normalized by dividing the amount of the mRNA of interest by that of *Gapdh* mRNA. Primers specific for mouse *Ins1*, *Ins2*, *Pdx1*, *Nkx2.2*, *MafA* and *Gapdh* were purchased as Assays-on-Demand Gene Expression Products (Applied Biosystems)^26^.

#### Glucose tolerance test

An intraperitoneal glucose tolerance test was performed on 6- and 12-week-old mice. The mice were fasted for 12 h and then were injected intraperitoneally (i.p.) with glucose (2.0 g/kg body weight). The blood glucose levels were measured before injection and at 5, 30, 60, 90 and 120 min after injection.

#### Statistics and reproducibility

Error bars indicate the standard error of at least three measurements. Student’s *t* tests were used to compare two samples from independent groups using Microsoft Excel. Repeated measures ANOVA was performed to compare data among groups. The differences between the groups were considered to be significant when *p* < 0.05.

All experiments were performed in accordance with relevant guidelines and regulations.

All methods were carried out in compliance with the ARRIVE guidelines.

## Supplementary Information

Supplementary Information.

## Data Availability

The data that support the findings of this study are available from the corresponding author, H.N., upon reasonable request.
