# Supplementary material for: In vivo evaluation of GG2–GG1/A2 element activity in the insulin promoter region using the CRISPR–Cas9 system
Source: Sci Rep. 2021 Oct 13;11:20290. doi: 10.1038/s41598-021-99808-6 (PMC8514523; doi:10.1038/s41598-021-99808-6)
Supplement: Supplementary file 1 — Supplementary Information. [file 41598_2021_99808_MOESM1_ESM.docx]

**Supplemental Information**

***In vivo* evaluation of the GG2-GG1/A2 element activity in the insulin promoter region using the CRISPR-Cas9 system**

Hirofumi Noguchi ^1,*^, Chika Miyagi-Shiohira ^1^, Takao Kinjo ^2^, Issei Saitoh ^3^, Masami Watanabe ^4^

1. Department of Regenerative Medicine, Graduate School of Medicine, University of the Ryukyus, Okinawa 903-0215, Japan

2. Department of Basic Laboratory Sciences, School of Health Sciences in Faculty of Medicine, University of the Ryukyus, Okinawa 903-0215, Japan

3. Department of Pediatric Dentistry, Asahi University School of Dentistry, Hozumi, 501-0296, Japan

4. Department of Urology, Okayama University Graduate School of Medicine, Dentistry and Pharmaceutical Sciences, Okayama 700-8558, Japan

**Supplemental tables/figures count:** tables 4/figures 3

**Supplementary Fig. 1. *Ins1* and *Ins2* mRNA levels in the embryonic pancreas of 1GG2GG mice.**

(A) qRT-PCR analysis of *Ins1* and *Ins2* in pancreatic islets of 1GG2GG male mice. (B) qRT-PCR analysis of *Ins1* and *Ins2* in pancreatic islets of 1GG2GG female mice.

**Supplementary Fig. 2. *Ins1* and *Ins2* mRNA levels of homozygous mice with a single mutation in the *Ins1* or *Ins2* promoter.**

qRT-PCR analysis of *Ins1* (**A, C**) and *Ins2* (**B, D**) in pancreatic islets of homozygous mice with a single mutation in the *Ins1* or *Ins2* promoter. Pancreatic islets (purity >95%) of wild-type mice served as a control. The data are expressed as the target gene to *Gapdh* ratio; that of the control cells was arbitrarily defined as 1 (n=8). **p*<0.05, ***p*<0.01

**Supplementary Fig. 3. Construction of the insulin promoter-luciferase plasmid.**

Wild-type or mutated *Ins1/2* promoter cDNAs containing approximately 1,000 bp of the 5´-flanking sequences of the *Ins1/2* promoter regions were ligated to luciferase plasmids using a ligation kit.

**Supplementary Table 1. Heterozygous mice with a deletion in the *Ins1* promoter or *Ins2* promoter**

| Name | Mutated Elements in *Ins1* Promoter | Mutated Elements in *Ins2* Promoter | Diabetes | Figure # | Comments |
| --- | --- | --- | --- | --- | --- |
| 1GG^+/-^ | GG2-GG1/A2 | none | - | Fig 2a | Deletion of elements bound by Nkx2.2 (Pdx1?) and A2.2 in *Ins1* promoter |
| 2GG^+/-^ | none | Mainly GG2 | - | Fig 2a | Deletion of elements bound by Nkx2.2 (Pdx1?) in *Ins2* promoter |
| 1C_1_^+/-^,1_2_^+/-^,  1_1_^+/-^,2_3_^+/-^,  2C_1_^+/-^,2_2_^+/-^,  2_1_^+/-^ | - | - | - | - | Ref. 22 |

**Supplementary Table 2. Homozygous mice with deletions in the *Ins1* promoter or *Ins2* promoter**

| Name | Mutated Elements in *Ins1* Promoter | Mutated Elements in *Ins2* Promoter | Diabetes | Figure # | Comments |
| --- | --- | --- | --- | --- | --- |
| 1GG | GG2-GG1/A2 | none | - | Fig 2b | Deletion of elements bound by Nkx2.2 (Pdx1?) and A2.2 in *Ins1* promoter |
| 2GG | none | Mainly GG2 | - | Fig 2b | Deletion of elements bound by Nkx2.2 (Pdx1?) in *Ins2* promoter |
| 1C_1_,1_2_,  1_1_,2_3_,  2C_1_,2_2_  2_1_ | - | - | - | - | Ref. 22 |

**Supplementary Table 3. Heterozygous mice with deletions in both the *Ins1* promoter and the *Ins2* promoter**

| Name | Mutated Elements in *Ins1* Promoter | Mutated Elements in *Ins2* Promoter | Diabetes | Figure # | Comments |
| --- | --- | --- | --- | --- | --- |
| 1GG2GG^+/-^ | GG2-GG1/A2 | Mainly GG2 | - | Fig 2a | Deletion of elements bound by Nkx2.2 (Pdx1?) and A2.2 in *Ins1* promoter  Deletion of elements bound by Nkx2.2 (Pdx1?) in *Ins2* promoter |
| 1GG2_3_^+/-^ | GG2-GG1/A2 | none | - | Fig 2a | Deletion of elements bound by Nkx2.2 (Pdx1?) and A2.2 in *Ins1* promoter |
| 1GG2C_1_^+/-^ | GG2-GG1/A2 | C (1 base only) | - | Fig 2a | Deletion of elements bound by Nkx2.2 (Pdx1?) and A2.2 in *Ins1* promoter |
| 1GG2_2_^+/-^ | GG2-GG1/A2 | none | - | Fig 2a | Deletion of elements bound by Nkx2.2 (Pdx1?) and A2.2 in *Ins1* promoter |
| 1GG2_1_^+/-^ | GG2-GG1/A2 | none | - | Fig 2a | Deletion of elements bound by Nkx2.2 (Pdx1?) and A2.2 in *Ins1* promoter |
| 1C_1_2GG^+/-^ | C (1 base only) | Mainly GG2 | - | Fig 2a | Deletion of elements bound by Nkx2.2 (Pdx1?) in *Ins2* promoter |
| 1_2_2GG^+/-^ | none | Mainly GG2 | - | Fig 2a | Deletion of elements bound by Nkx2.2 (Pdx1?) in *Ins2* promoter |
| 1_1_2GG^+/-^ | none | Mainly GG2 | - | Fig 2a | Deletion of elements bound by Nkx2.2 (Pdx1?) in *Ins2* promoter |
| 1C_1_2_3_^+/-^,  1C_1_2C_1_^+/-^,  1C_1_2_2_^+/-^  1C_1_2_1_^+/-^,  1_2_2_3_^+/-^,  1_2_2C_1_^+/-^,  1_2_2_2_^+/-^,  1_2_2_1_^+/-^,  1_1_2_3_^+/-^,  1_1_2C_1_^+/-^,  1_1_2_2_^+/-^,  1_1_2_1_^+/-^ | - | - | - | - | Ref. 22 |

**Supplementary Table 4. Homozygous mice with deletions in both the *Ins1* promoter and the *Ins2* promoter**

| Name | Mutated Elements in *Ins1* Promoter | Mutated Elements in *Ins2* Promoter | Diabetes | Figure # | Comments |
| --- | --- | --- | --- | --- | --- |
| 1GG2GG | GG2-GG1/A2 | Mainly GG2 | + | Fig 2b | Deletion of elements bound by Nkx2.2 (Pdx1?) and A2.2 in *Ins1* promoter  Deletion of elements bound by Nkx2.2 (Pdx1?) in *Ins2* promoter |
| 1GG2_3_ | GG2-GG1/A2 | none | - | Fig 2b | Deletion of elements bound by Nkx2.2 (Pdx1?) and A2.2 in *Ins1* promoter |
| 1GG2C_1_ | GG2-GG1/A2 | C (1 base only) | - | Fig 2b | Deletion of elements bound by Nkx2.2 (Pdx1?) and A2.2 in *Ins1* promoter |
| 1GG2_2_ | GG2-GG1/A2 | none | - | Fig 2b | Deletion of elements bound by Nkx2.2 (Pdx1?) and A2.2 in *Ins1* promoter |
| 1GG2_1_ | GG2-GG1/A2 | none | - | Fig 2b | Deletion of elements bound by Nkx2.2 (Pdx1?) and A2.2 in *Ins1* promoter |
| 1C_1_2GG | C (1 base only) | Mainly GG2 | - | Fig 2b | Deletion of elements bound by Nkx2.2 (Pdx1?) in *Ins2* promoter |
| 1_2_2GG | none | Mainly GG2 | - | Fig 2b | Deletion of elements bound by Nkx2.2 (Pdx1?) in *Ins2* promoter |
| 1_1_2GG | none | Mainly GG2 | - | Fig 2b | Deletion of elements bound by Nkx2.2 (Pdx1?) in *Ins2* promoter |
| 1C_1_2_3_,  1C_1_2C_1_,  1C_1_2_2_,  1C_1_2_1_,  1_2_2_3_,  1_2_2C_1_,  1_2_2_2_,  1_2_2_1_,  1_1_2_3_,  1_1_2C_1_,  1_1_2_2_,  1_1_2_1_ | - | - | - | - | Ref. 22 |
